# Viral strategies to antagonize the host antiviral innate immunity: an indispensable research direction for emerging virus-host interactions

**DOI:** 10.1080/22221751.2024.2341144

**Published:** 2024-06-07

**Authors:** Na Chen, Jiayu Jin, Baoge Zhang, Qi Meng, Yuanlu Lu, Bing Liang, Lulu Deng, Bingchen Qiao, Lucheng Zheng

**Affiliations:** a MOE Joint International Research Laboratory of Animal Health and Food Safety, Engineering Laboratory of Animal Immunity of Jiangsu Province, College of Veterinary Medicine, Nanjing Agricultural University, Nanjing, People’s Republic of China; b College of Veterinary Medicine, Nanjing Agricultural University, Nanjing, People’s Republic of China

**Keywords:** Emerging viruses, antagonism, host antiviral innate immunity, interferon production, interferon responses, SARS-CoV-2, IAV

## Abstract

The public's health is gravely at risk due to the current global outbreak of emerging viruses, specifically SARS-CoV-2 and MPXV. Recent studies have shown that SARS-CoV-2 mutants (such as Omicron) exhibit a higher capability to antagonize the host innate immunity, increasing their human adaptability and transmissibility. Furthermore, current studies on the strategies for MPXV to antagonize the host innate immunity are still in the initial stages. These multiple threats from emerging viruses make it urgent to study emerging virus-host interactions, especially the viral antagonism of host antiviral innate immunity. Given this, we selected several representative viruses that significantly threatened human public health and interpreted the multiple strategies for these viruses to antagonize the host antiviral innate immunity, hoping to provide ideas for molecular mechanism research that emerging viruses antagonize the host antiviral innate immunity and accelerate the research progress. The IAV, SARS-CoV-2, SARS-CoV, MERS-CoV, EBOV, DENV, ZIKV, and HIV are some of the typical viruses. Studies have shown that viruses could antagonize the host antiviral innate immunity by directly or indirectly blocking antiviral innate immune signaling pathways. Proviral host factors, host restriction factors, and ncRNAs (microRNAs, lncRNAs, circRNAs, and vtRNAs) are essential in indirectly blocking antiviral innate immune signaling pathways. Furthermore, via controlling apoptosis, ER stress, stress granule formation, and metabolic pathways, viruses may antagonize it. These regulatory mechanisms include transcriptional regulation, post-translational regulation, preventing complex formation, impeding nuclear translocation, cleavage, degradation, and epigenetic regulation.

## Introduction

The recent global outbreaks of SARS-CoV-2 and MPXV have had a significant impact on public health security worldwide. Recent data have shown that multiple factors contribute to the severity of COVID-19. Severe COVID-19 patients also had downregulated ISGs and decreased interferon production [1]. Notably, since its discovery in November 2021, the Omicron mutant strain has spread rapidly to about 150 countries and regions worldwide. Studies have found that Omicron, the most severe variant of SARS-CoV-2 so far, has evolved a greater ability to antagonize the host innate immunity, thereby increasing its human adaptability and transmissibility [2]. Monkeypox cases have been recorded nonstop since May 2022 in a number of non-African nations and areas. Monkeypox is a zoonotic disease caused by MPXV. More than 80,000 instances of monkeypox have been reported worldwide to date, according to the World Health Organization, in roughly 112 nations and territories. Of those cases, 116 have been deadly [3].

The public's health is at risk from a number of viruses and linked infectious diseases in addition to SARS-CoV-2 and MPXV. One common respiratory pathogen is the influenza virus. Seasonal influenza outbreaks kill between 250,000 and 500,000 people worldwide each year [4]. There are four types of influenza viruses: A, B, C, and D. Among all subtypes, IAV is the most common [5]. Besides, highly pathogenic coronaviruses also include SARS-CoV and MERS-CoV, which have been transmitted to humans and caused severe acute respiratory illness over the past two decades [6,7]. HIV-1 continues to be a major global public health concern as of right now, with over 40 million people infected and 20 million cases resulting in death worldwide [8]. Notably, these continuous outbreaks of infectious diseases caused by EBOV, DENV, and ZIKV have also caused significant threats to global health in the past few years. Between 60% and 90% of people who contract EBOV die from a severe hemorrhagic illness [9–11]. Furthermore, DENV is transmitted to humans via mosquitoes, resulting in about 400 million infections and tens of thousands of deaths yearly [12,13]. ZIKV has infected millions of individuals in the past ten years by causing significant epidemics in the tropics and spreading to other areas [14–17].

Researching the relationships between emerging viruses and hosts, particularly the viral antagonistic relationship with host antiviral innate immunity, is crucial given the numerous risks posed by emerging viruses such as SARS-CoV-2 and MPXV. We can only have a more comprehensive understanding of emerging viruses and their pathogenic mechanisms to quickly respond to each outbreak of mutant strains and global epidemics by accelerating the study's progress on emerging viral antagonism of host antiviral innate immunity. The mechanisms by which viruses antagonize the host antiviral innate immunity has been the subject of countless investigations over the last ten years, which have resulted in the formation of a rather organized theoretical framework. In this review, we selected several representative viruses that significantly threatened human public health and systematically elucidated the multiple strategies for these viruses or specific viral proteins to antagonize the host antiviral innate immunity, hoping to provide ideas for molecular mechanism research that emerging viruses antagonize the host antiviral innate immunity and accelerate the research progress. Current studies on the viral antagonism of host antiviral innate immunity have focused on the mechanisms of blocking antiviral innate immune signaling pathways. The primary regulatory mechanisms encompass transcriptional regulation, post-translational regulation (such as phosphorylation and K63-linked Ub), preventing complex formation, impeding nuclear translocation, cleavage, and degradation, and so forth. Among these, the autophagy and ubiquitin-proteasome pathways are involved in degradation.

## Block antiviral innate immune signaling pathways directly

**Figure 1.**
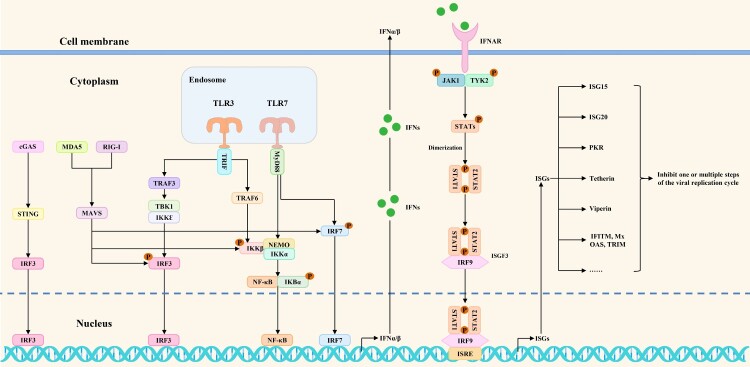
Antiviral innate immune signaling pathways mediated by RIG-I, MDA5, TLR3/7, and cGAS/STING. After virus infection of cells, antiviral innate immune signaling pathways are activated to induce the phosphorylation of transcription factors and IFNα/β expression. Subsequently, IFNα/β binds to interferon receptors on target cells, thereby initiating antiviral interferon responses and, ultimately, ISGs expression.

Emerging viral infections may trigger both innate and adaptive immune reactions. Innate immune responses are one of the current research hotspots in immunology. Antiviral innate immune signaling pathways play critical roles in innate immune responses. Pattern recognition receptors (PRRs) (such as cGAS/STING, MDA5, RIG-I, and TLR3/7) could recognize viral nucleic acids and activate transcription factors, including NF-κB, IRF3, and IRF7. During this process, MAVS, MyD88, TRAF3, TRAF6, TRIF, IκBα, TBK1/IKKϵ, and IKKα/IKKβ/NEMO complexes act as essential signaling proteins for signal transduction. These transcription factors cause the production of IFNα/β once they are activated. Following that, IFNα/β binds to interferon receptors and induces the phosphorylation of JAK1, TYK2, STAT1, and STAT2. Phosphorylated STAT1 and STAT2 bind to IRF9 and form ISGF3. The ISGF3 trimers then go to the nucleus, where they bind ISRE to cause the synthesis of ISGs, ultimately preventing the spread of the virus [5]. A schematic diagram of antiviral innate immune signaling pathways mediated by RIG-I, MDA5, TLR3/7, and cGAS/STING is shown in Figure 1. The battles between viruses and hosts have been constant. Host PRRs recognize viral nucleic acid and then rapidly activate antiviral innate immune signaling pathways to induce ISGs expression, ultimately blocking one or multiple steps of the viral replication cycle. In the meantime, viruses have developed a number of regulatory mechanisms that directly block antiviral innate immune signaling pathways. They could inhibit the expression of interferons and downstream ISGs by targeting and regulating PRRs, signaling proteins, transcription factors, and interferon responses. The initial exploration of these potential mechanisms is an indispensable research direction for emerging virus-host interactions. A summary of these regulatory mechanisms could provide clearer ideas for studying emerging virus-host interactions.

### Target PRRs

As PRRs, cGAS/STING, MDA5, and RIG-I are essential for innate immune responses. Nevertheless, viruses have evolved several strategies to affect how these PRRs work. For example, DENV NS2B targets the cGAS and degrades it in an autophagy-lysosome-dependent mechanism. Such degradation inhibits type I interferon production in the infected cell [12]. It has been demonstrated that SARS-CoV-2 Nsp5 inhibits K63-ubiquitin modification of STING to disrupt the assembly of the STING functional complex and downstream signaling. Furthermore, SARS-CoV-2 ORF3a could bind to STING and block the nuclear accumulation of p65, thereby inhibiting NF-κB signaling [18]. By contrast, DENV(NS2B3) and ZIKV(NS2B3) inhibit type I interferon production in infected cells by cleaving STING. It's interesting to note that the cytoplasmic loop of STING contains the residues R78 and G79, which are crucial factors in the cleavage that ZIKV NS2B3 mediates [15,19].

There are two types of ubiquitylation: degradative (like K48-linked polyubiquitination) and non-degradative (like K63-linked polyubiquitination). K63-linked polyubiquitination of RIG-I is critical for activating antiviral innate immune responses, whereas K48-linked polyubiquitination mediates RIG-I degradation. Several studies have shown that some viruses could rely on their proteins to interact with RIG-I and repress RIG-I-mediated IFN-β production through multiple pathways, such as inhibiting K63-linked polyubiquitination, preventing complex formation, and degradation, etc. These are IAV(NS1), SARS-CoV-2(M, N, and Nsp5), and ZIKV(NS5), among other viruses and viral proteins. Specifically, ZIKV NS5 may specifically bind to RIG-I by interaction with the CARD domain, which would limit K63-linked polyubiquitination of RIG-I, IRF3 activation, and IFN-β production, all of which would inhibit RIG-I signaling [20]. Besides, SARS-CoV-2 M interacts with RIG-I to impede the formation of the RIG-I-MAVS complex and inhibit IRF3 phosphorylation, thereby reducing type I and III interferon production [21]. Additionally, SARS-CoV-2 Nsp5 deprives RIG-I of its ability to activate MAVS by cleaving off the ten most N-terminal amino acids from RIG-I. In other words, Nsp5 cleaves RIG-I after the Q10 residue, preventing RIG-I from interacting with and activating MAVS [22]. Furthermore, through their interactions with RIG-I and inhibition of the RIG-I signaling pathway, IAV(NS1) and SARS-CoV-2(N) similarly repress IFN-β expression [23,24].

### Target signaling proteins

As a highly essential signaling protein, MAVS signaling is regulated by various viruses at multiple levels. These regulatory mechanisms encompass preventing complex formation and degradation, etc. For example, DENV NS4A interacts with MAVS to prevent RIG-I from forming complexes with MAVS, inhibiting RIG-I-induced IRF3 activation and IFN-β expression. The results show that the N-terminal CARD-like (CL) domain and the C-terminal transmembrane (TM) domain of MAVS contribute to the formation of the MAVS-NS4A complex [25]. Besides, ZIKV NS4A interacts with MAVS to prevent RLR from binding to MAVS, which suppresses type I interferons generated by RIG-I and MDA5 [17]. By contrast, the SARS-CoV-2 M protein interacts with MAVS to impair MAVS aggregation and its recruitment of downstream IRF3, TBK1, and TRAF3, attenuating the innate antiviral responses [26]. Studies have indicated that IAV PB1 and SARS-CoV-2 ORF10 could suppress the antiviral innate immune responses by degrading MAVS through autophagy pathways, while SARS-CoV-2 Nsp5 and ZIKV NS3 promote MAVS degradation via the ubiquitin-proteosome pathway [22,27–29]. IAV PB1-F2 and PB2 have been reported to interact with MAVS and inhibit MAVS-mediated IFN-β expression. Further research into the particular molecular regulatory mechanisms may be necessary [30,31].

A number of studies indicate that viruses may block signal transduction of other signaling proteins (including TRAF3, TBK1/IKKϵ, and IKKα/IKKβ/NEMO complexes) in addition to MAVS. These viruses may do this by a variety of mechanisms, such as post-translational regulation, preventing complex formation, and degradation, etc. Mechanistically, IAV NS1/126-225 interacts with TRAF3 to reduce K63-linked ubiquitination of TRAF3 and inhibit IRF3 phosphorylation, thereby decreasing IRF3-mediated IFN-β expression [32]. Besides, by impeding K63-linked polyubiquitination and TRAF3 activation, IAV PB2 may counteract the innate antiviral responses [33]. By contrast, MERS-CoV M interacts with TRAF3 and disrupts the TRAF3-TBK1 association, leading to reduced IRF3 activation [7]. As for TBK1/IKKϵ and IKKα/IKKβ/NEMO complexes, viruses also disrupt their signaling functions at various levels. Specifically, SARS-CoV-2(Nsp13) and ZIKV(NS5) could interact with TBK1 to inhibit its phosphorylation and impair the phosphorylation and nuclear translocation of IRF3, thereby reducing type I interferon production [34,35]. In addition, SARS-CoV-2 M may interact with TBK1 and induce its degradation via K48-linked ubiquitination. The reduced TBK1 further impairs the formation of the TRAF3-TANK-TBK1-IKKϵ complex, which leads to the inhibition of type I interferon production [36]. Furthermore, ZIKV NS5 binds to IKKϵ and inhibits its phosphorylation and expression, blocking IRF3 activation and interferon production [37]. Numerous investigations have demonstrated that by interacting with TBK1/IKKε and preventing complex formation, EBOV(VP35), MERS-CoV(ORF4b), and SARS-CoV(M) could inhibit IRF3 phosphorylation and IFN-β expression [9,38,39]. Additionally, the IAV NS1 protein specifically inhibits IKK-mediated NF-κB activation and the production of downstream antiviral genes by physically interacting with IKKα and IKKβ through the C-terminal effector domain [40]. Moreover, IAV PB1-F2 interacts with IKKβ to inhibit NF-κB binding to DNA and NF-κB signaling [41]. It's interesting to note that during viral infection, SARS-CoV-2 ORF9b is known to target the NEMO and disrupt its K63-linked polyubiquitination, which inhibits IKKα/β/γ-NF-κB signaling and subsequent interferon production [42].

### Target transcription factors and interferon responses

It is commonly recognized that the transcription factors NF-κB and IRF3 play a crucial role in regulating the expression of IFNα/β that is brought on by viral infections. However, the outcomes demonstrated that IAV, SARS-CoV, and SARS-CoV-2 could regulate the activity and nuclear translocation of IRF3 to inhibit IFNα/β expression. Mechanistically, IAV(PA) and SARS-CoV(PLpro) reduce IFN-β expression by inhibiting IRF3 phosphorylation [43,44]. By contrast, SARS-CoV(8b, 8ab) could target IRF3 and induce its degradation through ubiquitin-proteasome pathways [45]. Moreover, IAV(PA), SARS-CoV(PLpro), and SARS-CoV-2(Nsp5, Nsp12, and ORF3b) could suppress type I interferon production by interacting with IRF3 and preventing its nuclear translocation [43,44,46–48]. Viruses can target and modulate the nuclear translocation of NF-κB in addition to IRF3 in order to disrupt signaling pathways. IAV PA-X specifically inhibits NF-κB p65 nuclear translocation and NF-κB activity, which suppresses IFN-β production [49].

Once activated, transcription factors bind to the interferon promoter to regulate interferon expression and induce interferon responses. The interferon-activated JAK-STAT signaling pathway is essential for ISGs production. Nevertheless, several viruses could block it through a variety of mechanisms. According to reports, ZIKV NS2B3 promotes JAK1 degradation in a proteasome-dependent manner, hence inhibiting the JAK-STAT signaling pathway [50]. Multiple studies have shown that DENV, HIV-1, SARS-CoV-2, and ZIKV could inhibit the signaling of STAT1 and STAT2 to antagonize the host antiviral innate immunity. These antagonistic mechanisms include inhibiting phosphorylation and degradation. Specifically, SARS-CoV-2(Nsp13) interacts with STAT1 to inhibit its phosphorylation, whereas HIV-1(Vif) and SARS-CoV-2(Nsp5) promote its degradation through the ubiquitin-proteasome and autophagy pathways, respectively [6,8,51]. In addition, DENV(NS5) and ZIKV(NS5) suppress interferon signaling by inducing STAT2 degradation via the ubiquitin-proteasome pathways [13,14]. Besides, SARS-CoV-2 N could block interferon responses by suppressing the phosphorylation of STAT1 and STAT2 [52]. By contrast, ZIKV(NS2A) degrades both STAT1 and STAT2 through ubiquitin-proteasome pathways [16]. Studies have indicated that SARS-CoV-2 N, ORF6, and ORF8 inhibit the ISRE activation induced by SeV infection to suppress interferon signaling [52,53]. PKR, an ISG, detects viral RNA in the cytoplasm and phosphorylates eIF2α to inhibit viral protein synthesis and viral replication. However, EBOV(VP35), IAV (NS1), and MERS-CoV(ORF4a) could block PKR activation to antagonize the host antiviral innate immunity. Mechanistically, EBOV VP35 could antagonize PKR activity through its C-terminal interferon inhibitory domain [10]. Furthermore, the results of the experiments demonstrate that PKR activation is inhibited by direct PKR binding to the IAV NS1. The NS1 amino acid sequence (123–127) is necessary to inhibit PKR activation in virus-infected cells [54]. Additionally, MERS-CoV ORF4a suppresses PKR-mediated translation inhibition through its dsRNA-binding domain [55]. The viral strategies to directly block multiple steps of antiviral innate immune signaling pathways are summarized in Table 1. This summary might help us better understand the relationships among viruses (viral proteins), regulated targets, and steps of blocked signaling pathways.

**Table 1.** The viral strategies to directly block multiple steps of antiviral innate immune signaling pathways.

Steps of blocked signaling pathways	Targets	Viruses and their proteins	Ref.
PRRs	cGAS	DENV(NS2B)	[12]
	RIG-I	IAV(NS1)	[23]
		SARS-CoV-2(M, N, and Nsp5)	[21,22,24]
		ZIKV(NS5)	[20]
	STING	DENV(NS2B3)	[19]
		SARS-CoV-2(Nsp5, ORF3a)	[18]
		ZIKV(NS2B3)	[15]
Signaling proteins	MAVS	DENV(NS4A)	[25]
		IAV(PB1, PB1-F2, and PB2)	[27,30,31]
		SARS-CoV-2(M, Nsp5, and ORF10)	[22,26,28]
		ZIKV(NS3, NS4A)	[17,29]
	TRAF3	IAV(NS1, PB2)	[32,33]
		MERS-CoV(M)	[7]
	TBK1	SARS-CoV-2(M, Nsp13)	[34,36]
		ZIKV(NS5)	[35]
	IKKϵ	ZIKV(NS5)	[37]
	TBK1/IKKϵ	EBOV(VP35)	[9]
		MERS-CoV(ORF4b)	[38]
		SARS-CoV(M)	[39]
	IKKα	IAV(NS1)	[40]
	IKKβ	IAV(NS1, PB1-F2)	[40,41]
	NEMO	SARS-CoV-2(ORF9b)	[42]
Transcription factors	IRF3	IAV(PA)	[43]
		SARS-CoV(8b, 8ab, and PLpro)	[44,45]
Nuclear translocation of transcription factors	IRF3	IAV(PA)	[43]
		SARS-CoV(PLpro)	[44]
		SARS-CoV-2(Nsp5, Nsp12, and ORF3b)	[46–48]
	NF-κB	IAV(PA-X)	[49]
Interferon responses	JAK1	ZIKV(NS2B3)	[50]
	STAT1	HIV-1(Vif)	[8]
		SARS-CoV-2(N, Nsp5, and Nsp13)	[6,51,52]
		ZIKV(NS2A)	[16]
	STAT2	DENV(NS5)	[13]
		SARS-CoV-2(N)	[52]
		ZIKV(NS2A, NS5)	[14,16]
	ISRE activity	SARS-CoV-2(N, ORF6, and ORF8)	[52,53]
	PKR	EBOV(VP35)	[10]
		IAV(NS1)	[54]
		MERS-CoV(ORF4a)	[55]

## Block antiviral innate immune signaling pathways indirectly by hijacking ncRNAs and proviral host factors or antagonizing host restriction factors

Host ncRNAs are well established to be essential regulators of host biological processes, including apoptosis, inflammation, and viral replication [56]. It has been demonstrated that viruses could also antagonize the host antiviral innate immunity by upregulating the expression of microRNAs, lncRNAs, circRNAs, and vtRNAs to block multiple steps of antiviral innate immune signaling pathways indirectly. “Hijacking” refers to the positive regulatory effects of viruses on the expression of certain host biomolecules.

MicroRNAs are a kind of ncRNAs with approximately 20 nucleotides in length. According to a number of studies, when viruses seize control of microRNAs, they typically attach to the 3′ untranslated regions (3′UTRs) of the target genes to prevent transcription and protein synthesis, which in turn prevents signal transduction. For example, miR-485 suppresses RIG-I expression after viral infection by directly binding to its 3′UTR. Further studies found that the suppression was mediated through posttranscriptional silencing by degrading the RIG-I mRNA. Besides, this interaction substantially reduces the RIG-I-mediated antiviral response, promoting IAV replication [56]. Furthermore, Li et al. discovered that IAV infection could trigger the synthesis of miR-125a, which inhibits the translation of MAVS mRNA by binding to a functional location on its 3′-UTR. Further studies indicated that inhibition of MAVS expression by miR-125a reduces IFN-β responses, leading to increased viral replication [57]. Remarkably, the outcomes of the experiments indicate a considerable rise in miR-146a expression following viral infections. Moreover, overexpression of miR-146a promoted DENV and IAV replication, while downregulation of miR-146a repressed replication. Further investigation revealed that the proviral role of miR-146a was mediated by targeting TRAF6 and inhibiting IFN-β production. These findings suggested that miR-146a may be a potential therapeutic target in viral infections since DENV and IAV could hijack it to target TRAF6 and reduce its expression, inhibiting interferon production [58,59].

Long noncoding RNA (lncRNA) is a class of ncRNA greater than 200 nucleotides in length. Numerous biological processes, including host antiviral responses and IAV replication, are regulated by lncRNAs, according to studies. Besides, it has been found that IAV could hijack lncRNAs to block antiviral innate immune signaling pathways indirectly. Specifically, Jiang et al. discovered that during IAV infection, monocytes had significant levels of lncRNA-NSPL expression. Furthermore, NSPL overexpression makes animals more prone to IAV infection than WT mice, while NSPL knockdown dramatically reduces IAV replication in THP-1 cells. Additional research revealed that by binding to RIG-I and obstructing the interaction between RIG-I and TRIM25, lncRNA-NSPL might impede TRIM25-mediated K63-linked ubiquitination of RIG-I and inhibit antiviral innate immune responses [60]. In addition, Li et al. identified a lncRNA named lncRNA-MxA, which is upregulated after IAV infection. They found that the lncRNA-MxA overexpression facilitates IAV replication, while lncRNA-MxA knockdown inhibits it. The IFN-β promoter may form triplexes with lncRNA-MxA, which would prevent NF-κB and IRF3 from binding to the promoter and hence inhibit the transcription of IFN-β, according to more research. These findings show that IAV may induce lncRNA-MxA expression to inhibit IFN-β transcription and antagonize the host antiviral innate immunity [61]. Additionally, Wang et al. observed that lncRNA-TSPOAP1-AS1 was significantly induced in A549 cells after IAV infection and poly (I:C) stimulation. Interestingly, TSPOAP1-AS1 induction by IAV infection was regulated by the NF-κB signaling pathway. Subsequent research revealed that TSPOAP1-AS1 promotes IAV replication by suppressing ISRE activation and the production of downstream ISGs [62].

**Figure 2.**
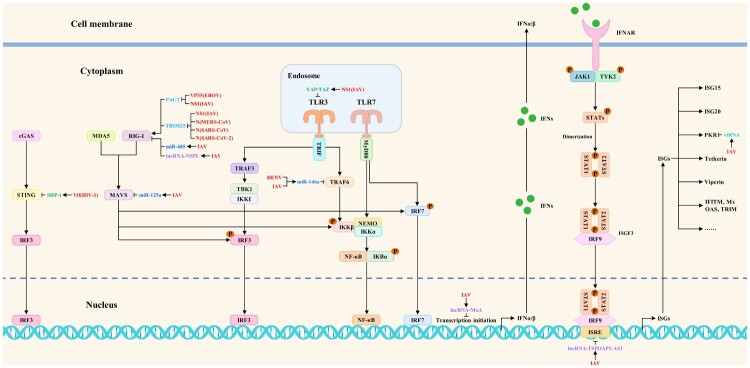
The viral strategies to indirectly block antiviral innate immune signaling pathways by hijacking ncRNAs and proviral host factors or antagonizing host restriction factors. Red, blue, purple, peacock blue, green, and light blue indicate viral proteins (viruses), microRNAs, lncRNAs, vtRNAs, proviral host factors, and host restriction factors, respectively.

Circular RNAs (circRNAs) are a class of ncRNA molecules that lack a 5′ end cap and a 3′ terminal poly(A) tail. Interestingly, Qiu et al. identified one circRNA (circ-MerTK) whose expression was upregulated during IAV infection. The findings showed that, in comparison to the empty vector control during infection, circ-MerTK overexpression reduced the mRNA levels of Mx1, ISG15, and IFN-β. Besides, NIH/3T3 cells expressing sh-circMerTK increased the expression of Mx1, IFITM3, and IFN-β, suggesting that IAV infection could induce circ-MerTK expression to regulate the expression of IFN-β and some ISGs negatively, thereby enhancing viral replication [63]. The vault RNAs (vtRNAs) are a class of 84-141-nt long eukaryotic ncRNAs that RNA polymerase III transcribes. As of right now, vtRNA1-1, vtRNA1-2, vtRNA1-3, and vtRNA2-1 are the four vtRNAs that have been found in human cells. IAV infection induces the host vtRNAs expression through its NS1 protein, as discovered by Li et al. The vtRNAs, hijacked by IAV, enhance viral replication by inhibiting the activation of the antiviral protein PKR and the subsequent interferon expression [64]. The viral strategies to indirectly block antiviral innate immune signaling pathways by hijacking ncRNAs are summarized in Figure 2.

Host factors related to viral replication include proviral host factors and host restriction factors. Proviral host factors directly or indirectly promote viral replication, whereas host restriction factors inhibit it. In addition to upregulating the expression of ncRNAs, viruses could also antagonize the host antiviral innate immunity by hijacking proviral host factors or antagonizing host restriction factors to block multiple steps of antiviral innate immune signaling pathways indirectly. As a proviral host factor, SHP-1 could interact with STING to inhibit K63-linked ubiquitination of STING, thereby suppressing type I interferon production. To dampen the antiviral responses, the association of HIV-1 Vif with SHP-1 facilitates SHP-1 recruitment to STING and inhibits the K63-linked ubiquitination of STING by dephosphorylating STING at Tyr162 [65]. Interestingly, IAV NS1 hijacks YAP/TAZ to suppress the TLR3-mediated innate immune responses. Mechanistically, NS1 could activate YAP/TAZ by direct interaction with YAP/TAZ through their C-terminal TAD. TLR3 expression is downregulated by the activated YAP/TAZ, which inhibits the antiviral innate immune signaling pathways [66]. These studies extend the research direction on the molecular mechanisms by which viruses antagonize the host antiviral innate immunity. The viral strategies to indirectly block antiviral innate immune signaling pathways by hijacking proviral host factors are summarized in Figure 2.

As a host restriction factor, TRIM25 consists of a SPRY domain, a RING-finger domain, a coiled-coil dimerization domain, and two B-box domains. The SPRY domain interacts with RIG-I CARDs, and the RING-finger domain with E3 ligase activity mediates Lys 63-linked ubiquitination of the N-terminal CARDs of RIG-I, thereby activating the RIG-I pathway and increasing interferon production [67]. Nonetheless, it has been reported that IAV(NS1), MERS-CoV(N), SARS-CoV(N), and SARS-CoV-2(N) could impair the interaction of TRIM25 with RIG-I to inhibit TRIM25-mediated RIG-I ubiquitination and indirectly block antiviral innate immune signaling pathways. Mechanistically, RIG-I signal transduction could be suppressed by IAV NS1 by inhibiting TRIM25-mediated RIG-I CARD ubiquitination. When NS1 interacts with the coiled-coil domain of TRIM25, it blocks TRIM25 multimerization and RIG-I CARD ubiquitination. This connection is mediated by residues E96 and E97 [68]. Additionally, the MERS-CoV N competes with RIG-I for interaction with TRIM25, thereby inhibiting K63-linked ubiquitination of RIG-I CARDs and RIG-I-mediated type I interferon induction [67]. Furthermore, the C terminus of the SARS-CoV N protein interacts with the TRIM25 SPRY domain to inhibit TRIM25-mediated RIG-I ubiquitination and interferon production by preventing the association between TRIM25 and RIG-I [69]. Moreover, SARS-CoV-2 N could also block antiviral innate immune signaling pathways by interacting with the TRIM25 SPRY domain and inhibiting TRIM25-mediated RIG-I activation [70]. PACT is a double-stranded RNA-binding protein that has been shown to interact with the carboxy-terminal domain of RIG-I, promoting RIG-I signaling and host interferon responses. Besides, it could also inhibit viral RNA synthesis and EBOV replication, suggesting that PACT is an essential regulator of RIG-I-mediated signaling pathways and viral replication [11]. Interestingly, research has suggested that EBOV(VP35) and IAV(NS1) may directly interact with PACT to prevent PACT from interacting with and activating RIG-I, therefore counteracting RIG-I-mediated interferon responses [11,71]. The counteracting effects of viruses on host antiviral responses are called “antagonizing”. The viral strategies to indirectly block antiviral innate immune signaling pathways by antagonizing host restriction factors are summarized in Figure 2. These mechanisms summarized above may provide new perspectives for studying emerging virus-host interactions.

## Regulation of metabolic pathways, stress granule formation, apoptosis, and ER stress

In addition to directly or indirectly blocking antiviral innate immune signaling pathways, viruses also antagonize the host antiviral innate immunity by regulating metabolic pathways, the formation of stress granules, apoptosis, and ER stress. These mechanisms might also be one of the research hotspots in emerging virus-host interactions. Specifically, lactate dehydrogenase A (LDHA) expression and LDHA-mediated lactate production were observed to be elevated in response to IAV infection by Thyrsted et al. Interestingly, lactate promotes IAV replication by inhibiting MAVS-dependent induction of type I interferons in primary human airway epithelium. These results indicated that IAV infection could induce lactate formation, which would counteract host antiviral innate defense by reducing interferon production and ISGs expression [72]. Besides, as the omega-3 polyunsaturated fatty acid (PUFA)-derived lipid mediator, protectin D1 (PD1) is reported to reduce IAV replication by inhibiting nuclear export of viral transcripts. Remarkably, during IAV infection, there was a substantial inhibition in PD1 production [73]. Furthermore, Singh et al. showed that AMPK activation has an antiviral effect and inhibits virus-induced glycolysis. In-depth studies revealed that ZIKV could antagonize the host antiviral innate immunity by reducing AMPK activation [74]. One potential way to treat patients with critical diseases might be to target these metabolic alterations. In response to environmental stimuli, including heat shock, oxidative stress, malnutrition, and viral infections, eukaryotic cells can initiate a cascade of reactions. One such response is the formation of stress granules (SGs) to help the cells cope with environmental pressures. Viral infections are one of the conditions for inducing the formation of SGs. The viruses might employ the host translational machinery to complete their life cycles after invading the host cell. Nevertheless, in order to create SGs and fend off viral invasion, the hosts might pause translation. Scientists’ interest in the connection between SGs and antiviral innate immunity has grown during the past few years. During the study, it was surprisingly found that SARS-CoV-2, SARS-CoV, MERS-CoV, and ZIKV could rely on their proteins to inhibit the formation of SGs and promote viral replication [75]. For example, following a coronavirus infection, the viral dsRNA binds to PKR and promotes PKR autophosphorylation, dimerization, and oligomerization. The phosphorylation of eIF2α by the active PKR triggers the recruitment of proteins that nucleate SGs, namely G3BP1, and ultimately results in the assembly of SGs. Nonetheless, the NPs of SARS-CoV-2, SARS-CoV, and MERS-CoV could inhibit PKR phosphorylation and impair SGs formation, promoting a cellular environment propitious for viral replication [75]. Moreover, ZIKV has been shown to inhibit SGs assembly in a phospho-eIF2α-dependent way, which counteracts host antiviral stress responses to enhance viral replication [76].

One of the main mechanisms of programmed cell death connected to the development and etiology of viral diseases is apoptosis. It is essential for viral replication and propagation. Studies have shown that API5, an anti-apoptotic protein, inhibits E2F1-dependent apoptotic signaling and IAV replication. To facilitate E2F1-dependent apoptosis, IAV NP may, nevertheless, repress API5 expression, hence preserving viral replication and dissemination [77]. The endoplasmic reticulum is responsible for protein synthesis, processing, and maturation within the cell. When infecting cells, viruses also utilize the endoplasmic reticulum to complete viral protein synthesis. Simultaneously, the build-up of substantial viral protein quantities triggers a stress response in the endoplasmic reticulum, which subsequently regulates several signaling pathways to preserve cellular homeostasis, leading to the development of autophagy, apoptosis, and metabolic syndrome [78]. Curiously, viruses might have evolved strategies to reduce the inevitable ER stress to a level that is advantageous for viral replication. For example, IAV NS1 interferes with the messenger RNA processing factor CPSF30 and suppresses ER stress response factors, such as XBP1, thereby antagonizing ER stress induction and promoting viral replication [78]. These results provide great ideas for further studying the viral antagonism of host antiviral innate immunity.

## Emerging viral strategies to antagonize the host antiviral innate immunity: several future research directions

Research on viral antagonistic mechanisms, as it stands, advances our knowledge of emerging viruses and their pathogenic mechanisms. Therefore, viral strategies to antagonize the host antiviral innate immunity are an indispensable research direction for emerging virus-host interactions. The question of how to investigate the antagonistic mechanisms of emerging viruses more thoroughly and quickly is coming into focus. The most likely solution, without a doubt, is to enhance the learning of research ideas among different viruses. It could provide essential inspiration for studying mechanisms by which emerging viruses antagonize the host antiviral innate immunity. Given this, we try to propose several research directions for SARS-CoV-2, MPXV, and future unknown emerging viruses to antagonize the host antiviral innate immunity based on these summarized viral strategies, which is expected to accelerate the progress of research on emerging viruses to antagonize the host antiviral innate immunity and to deepen the understanding of the pathogenic mechanisms of emerging viruses.

Over the past few years, many studies have been carried out worldwide to investigate the molecular mechanisms by which emerging SARS-CoV-2 antagonizes host antiviral innate immunity. Nevertheless, we find that the molecular mechanism studies that SARS-CoV-2 antagonizes host antiviral innate immunity are often limited to direct regulation of innate immune signaling pathways by viral proteins. Furthermore, the antagonistic mechanisms involving proviral host factors, host restriction factors, ncRNAs (such as lncRNAs, microRNAs, circRNAs, and vtRNAs), metabolic pathways, apoptosis, and ER stress are hardly studied. By contrast, the mechanisms of IAV in these aspects mentioned above are relatively mature, which could provide many important research ideas [56,62–64,66,68,72,77,78]. Additionally, using confocal microscopy, Yi et al. discovered that the PA protein of IAV interacts with IRF3 in the cytoplasm and prevents its phosphorylation, with the N-terminal domain of PA protein playing a crucial role in this process. Further studies revealed that the binding activity of PA protein to IRF3 depends on Asp108, the 108th amino acid in PA protein, and mutation of this aspartate site could weaken the inhibitory effect on IFN-β expression [43]. This discovery provides a substantial reference value for investigating the molecular mechanisms by which SARS-CoV-2 antagonizes host antiviral innate immunity, expanding the research idea of subcellular localization, structural domains, and binding sites of viral-host protein interactions.

MPXV, a member of the Poxviridae family and genus Orthopoxvirus, is the causative agent of monkeypox. MPXV, another representative of emerging viruses, gravely threatens global health security. Worryingly, the current studies on the strategies for MPXV to antagonize the host antiviral innate immunity are still in the initial stages. Some inferences are often drawn from studies performed with the vaccinia virus and related orthopoxviruses [3]. Nevertheless, the lack of a sufficiently structured theoretical underpinning emerging from these research results has caused the MPXV study to proceed slowly. Therefore, the viral strategies to antagonize the host antiviral innate immunity summarized above could also provide new ideas for studying emerging MPXV. For example, from Table 1, we discovered that viral strategies directly blocking antiviral innate immune signaling pathways have two crucial features. Multiple viral proteins could work together to antagonize one host factor or signal transduction process. Additionally, one single viral protein may antagonize multiple host factors or signal transduction processes. Given this, we speculated whether some MPXV proteins could synergistically antagonize the same host factor or signal transduction process. Furthermore, screening for MPXV proteins that can antagonize multiple signal transduction processes may be worthwhile. In addition to the connections between viruses (viral proteins), regulated targets, and steps of blocked signaling pathways, the antagonistic mechanisms should not be ignored. In other words, the mechanisms by which viruses or viral proteins regulate their targets mainly include transcriptional regulation, post-translational regulation, preventing complex formation, impeding nuclear translocation, cleavage, and degradation, etc. Which mechanism MPXV employs deserves further study. The current studies on the strategies for MPXV (a DNA virus) to antagonize the host antiviral innate immunity are still in the initial stages. Consequently, the relevant research results of RNA viruses (e.g. IAV and SARS-CoV-2) could provide many new ideas and references for MPXV studies. Borrowing ideas from other viruses (not limited to the vaccinia virus and related orthopoxviruses) could accelerate the progress of the MPXV study.

Undoubtedly, exploring new mechanisms by which emerging viruses antagonize the host antiviral innate immunity is essential because it could help us understand the pathogenic mechanisms of emerging viruses more comprehensively. Based on the mechanisms summarized above, we hypothesize that it may be traditional research ideas to explore how they directly block antiviral innate immune signaling pathways when facing unknown emerging viruses in the future. Consequently, whether emerging viruses could indirectly block antiviral innate immune signaling pathways by hijacking ncRNAs and proviral host factors or antagonizing host restriction factors deserves further study to expand the research direction. Furthermore, whether emerging viruses antagonize the host antiviral innate immunity by regulating metabolic pathways, the formation of stress granules, apoptosis, and ER stress also deserves further exploration. In addition, it is worth pondering which viral proteins regulate each of these processes that antagonize the host antiviral innate immunity and their regulatory mechanisms. In summary, the relatively systematic mechanisms outlined above are expected to accelerate the study's progress on the antagonistic effects of emerging SARS-CoV-2 and MPXV on host antiviral innate immunity, as well as provide a wealth of new research ideas for emerging virus-host interactions. Additionally, it could enable us to more effectively handle future public health threats posed by unknown emerging viruses.

## Concluding remarks and future perspectives

**Figure 3.**
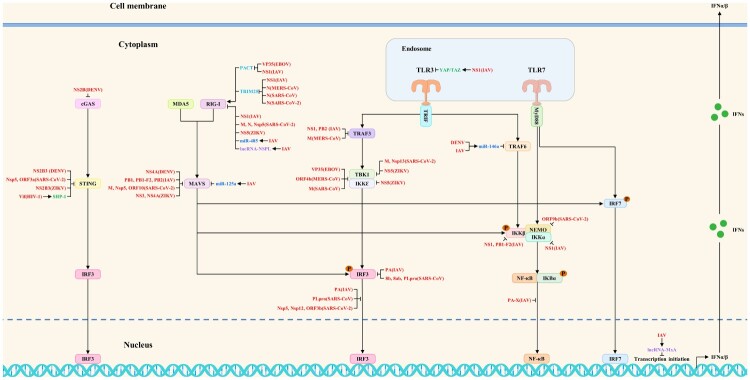
The viral strategies to block antiviral innate immune signaling pathways: inhibition of interferon induction. Viruses could block interferon production by targeting and regulating multiple steps of antiviral innate immune signaling pathways such as PRRs, signaling proteins, and transcription factors. Red, blue, purple, green, and light blue indicate viral proteins (viruses), microRNAs, lncRNAs, proviral host factors, and host restriction factors, respectively.

**Figure 4.**
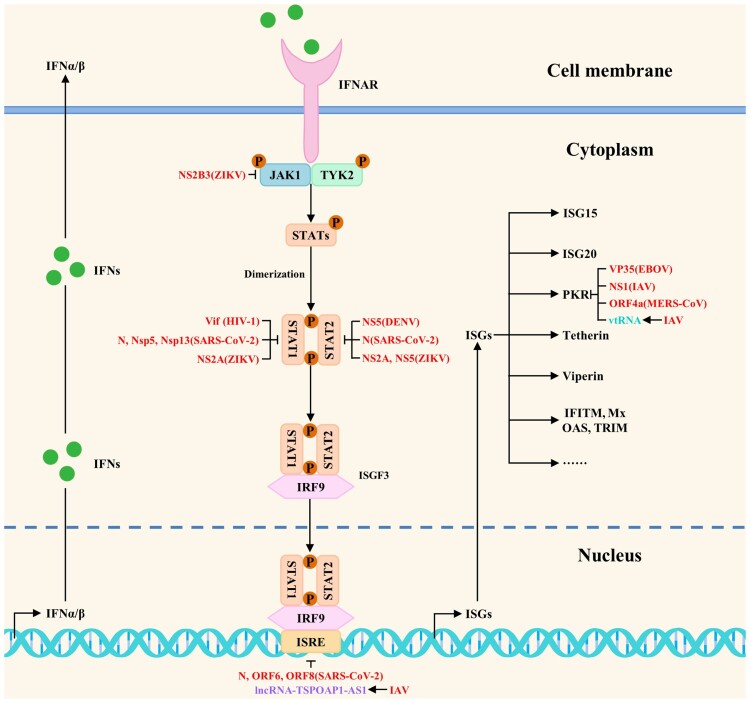
The viral strategies to block antiviral innate immune signaling pathways: inhibition of interferon responses. Viruses could antagonize the host antiviral innate immunity by directly or indirectly blocking interferon responses. Red, purple, and peacock blue indicate viral proteins (viruses), lncRNAs, and vtRNAs, respectively.

Studies have shown that viruses have evolved multiple strategies to antagonize the host antiviral innate immunity by directly or indirectly blocking the host innate immune signaling pathways during the long game with hosts. The related host proteins include proviral host factors and host restriction factors, whereas the ncRNAs include microRNAs, lncRNAs, circRNAs, and vtRNAs. The viral strategies to block multiple steps of antiviral innate immune signaling pathways are shown in Figure 3 and Figure 4. In addition, viruses also antagonize the host antiviral innate immunity by regulating metabolic pathways, the formation of stress granules, apoptosis, and ER stress. The current studies on viral strategies to antagonize the host antiviral innate immunity mainly focus on blocking multiple steps of host innate immune signaling pathways, including targeted inhibition of PRRs, signaling proteins, transcription factors, and interferon responses. Besides, we try to propose several research directions for SARS-CoV-2, MPXV, and future unknown emerging viruses to antagonize the host antiviral innate immunity based on these summarized viral strategies.

Interestingly, viruses can antagonize the host antiviral innate immunity in ways other than the ones listed above. For instance, Timilsina et al. show that SERINC5 could prevent viral fusion during entry, suggesting it is a host restriction factor for SARS-CoV-2. By preventing SERINC5 from being incorporated into progeny virions, SARS-CoV-2 ORF7a can decrease its antiviral function, according to more research. In addition, stimulation with IFN-β does not affect SERINC5 expression levels [79]. These results suggest that SARS-CoV-2 could antagonize a non-ISG host restriction factor, which exerts its antiviral effect by directly targeting the viral replication cycle rather than the antiviral innate immune signaling pathways mentioned above. Besides, studies have shown that IAV NS1 could interact with DNMT3B and promote its K48-linked ubiquitination and degradation. Next, the methylated promoter regions of the JAK-STAT signaling suppressors undergo fast demethylation, thereby increasing their expression and inhibiting JAK-STAT signaling [80]. This study involving epigenetic regulation enriches the understanding of mechanisms by which viruses antagonize the host antiviral innate immunity by indirectly blocking antiviral innate immune signaling pathways. Interestingly, the expression of miR-1249, miR-1307-3p, miR-584-5p, miR-324-5p, and miR-491 is downregulated during viral infections despite the fact that these molecules may directly inhibit IAV replication by targeting and decreasing the expression of viral proteins [5]. These studies further expand the targets of IAV antagonism from host restriction factors to ncRNAs. Beyond that, there may be more new antagonistic mechanisms to be studied and summarized in the future. There needs to be more analysis and discussion of future research trends regarding the viral antagonism of host antiviral innate immunity (Box 1).

Box 1. **Future research trends regarding the viral antagonism of host antiviral innate immunity**

Based on previous research results, borrowing research ideas of known antagonistic mechanisms among different viruses, further study of known antagonistic mechanisms, and exploring unknown antagonistic mechanisms may be one of the future research trends regarding the viral antagonism of host antiviral innate immunity. Although current studies on the mechanism by which viruses antagonize the host antiviral innate immunity have formed a relatively systematic theoretical system, there is still a certain gap in the research progress on antagonistic mechanisms among different viruses. For example, there are relatively few research results for EBOV to antagonize the host antiviral innate immunity compared to other representative viruses. Consequently, it is necessary to strengthen the borrowing of research ideas about known antagonistic mechanisms among different viruses. Specifically, more antagonistic mechanisms by which EBOV antagonizes the host antiviral innate immunity by directly blocking antiviral innate immune signaling pathways need to be further expanded. Besides, it remains to be answered whether its antagonistic mechanism is related to proviral host factors, host restriction factors, ncRNAs (such as lncRNAs, microRNAs, circRNAs, and vtRNAs), metabolic pathways, apoptosis, and ER stress. According to the research ideas mentioned above, the antagonistic mechanisms of other viruses could be further explored based on their respective research progress. Furthermore, some studies have not yet elucidated which viral protein(s) mediate viral antagonism of host antiviral innate immunity. They deserve further study, which may deepen the understanding of viral antagonistic mechanisms and provide a theoretical basis for developing antiviral drugs based on these further investigated antagonistic mechanisms in the future [5].

Notably, significant progress has been made in the past few years in elucidating the molecular mechanisms by which different viruses antagonize the host antiviral innate immunity. Nevertheless, there are still several knowledge gaps to be further investigated. Previous studies have shown that host biomolecules associated with viral replication include proteins, ncRNAs, sugars, lipids, hormones, and inorganic salts. In addition to the proteins, ncRNAs, sugars, and lipids mentioned above, it remains to be investigated whether other biomolecules are intrinsically linked to viral antagonism of host antiviral innate immunity [5]. If so, how do viruses regulate them? With the rapid development of single-cell technologies, the mechanisms of virus-host interactions are beginning to be explored at the single-cell level [5]. It is challenging to elucidate the molecular mechanisms by which different viruses antagonize the host antiviral innate immunity at the single-cell level, especially the regulation mechanisms between viruses and host biomolecules (not limited to proteins). Finally, it will be an enormous challenge for future researchers to explore the viral dynamic antagonism of host antiviral innate immunity, especially the dynamic interactions between viral proteins and host biomolecules. These will be interesting topics for future research. Moving forward, we must constantly extend our knowledge of viral strategies to antagonize the host antiviral innate immunity.

The World Health Organization has recently called for preparedness to combat the next wave of widespread infectious diseases brought on by unknown viruses or pathogens and has been researching the likelihood of a devastating pandemic brought on by an unknown disease. Given this, mechanism studies and summarization of strategies for different viruses to antagonize the host antiviral innate immunity are necessary. In other words, there is an urgent need to constantly extend our knowledge of viral strategies to antagonize the host antiviral innate immunity. In this manner, when confronted with the next emerging viral outbreak, we may quickly conduct investigations of the pertinent molecular mechanisms and draw on prior research to obtain a more thorough understanding of emerging viruses and their pathogenic mechanisms. In this manner, we may continue to defend human public health with knowledge and promptly respond to public health threats from unknown emerging viruses in the future.

## Glossary

**Table:** 

SARS-CoV-2	Severe acute respiratory syndrome coronavirus 2
MPXV	Monkeypox virus
IAV	Influenza A virus
SARS-CoV	Severe acute respiratory syndrome coronavirus
MERS-CoV	Middle East respiratory syndrome
EBOV	Ebola virus
DENV	Dengue virus
ZIKV	Zika virus
HIV	Human immunodeficiency virus
ncRNAs	Noncoding RNAs
ER	Endoplasmic reticulum
COVID-19	Coronavirus Disease 2019
ISGs	IFN-stimulated genes
cGAS	Cyclic GMP-AMP synthase
STING	Stimulator of interferon genes
MDA5	Melanoma differentiation-associated gene 5
RIG-I	Retinoic acid-inducible gene-I
TLR3/7	Toll-like receptor 3/7
NF-κB	Nuclear factor κappa-light-chain-enhancer of activated B cells
IRF3/7/9	Interferon regulatory factor 3/7/9
MAVS	Mitochondrial antiviral signaling
MyD88	Myeloid differentiation factor 88
TRAF3/6	TNF receptor-associated factor 3/6
TRIF	Toll/interleukin-1 (IL-1) receptor domain-containing adaptor inducing IFN-β
IκBα	The alpha inhibitor of NF-κB
TBK1	TANK-binding kinase 1
IKKs	IkappaB kinases
NEMO	NF-κB essential modulator
IFNα/β	Interferon α/β
JAK1	Janus kinase 1
TYK2	Tyrosine kinase 2
STATs	Signal transducers and activators of transcriptions
ISGF3	IFN-stimulated gene factor 3
ISRE	IFN-stimulated response elements
RLR	RIG-I like receptor
PKR	Protein kinase R
eIF2α	Eukaryotic initiation factor 2α
Mx1	Myxovirus resistance 1
ISG15	Interferon-stimulated gene 15
IFITM3	Interferon-induced transmembrane protein 3
SHP-1	Src homology 2-containing tyrosine phosphatase 1
YAP/TAZ	Yes-associated protein/ Transcriptional co-activator with PDZ-binding motif
TRIM25	Tripartite motif protein 25
PACT	PKR activator
AMPK	AMP-activated protein kinase
SGs	Stress granules
G3BP1	GTPase-activating protein SH3 domain-binding protein 1
API5	Apoptosis inhibitor 5
E2F1	E2F transcription factor 1
CPSF30	Cleavage and polyadenylation specificity factor 30
XBP1	X-box binding protein 1
SERINC5	Serine incorporator 5
DNMT3B	DNA methyltransferase 3B
